# The Situation and Factors Associated with Dental Caries Among Preschool Children in Nakhon Si Thammarat Province, Thailand

**DOI:** 10.3390/children13091282

**Published:** 2026-09-21

**Authors:** Pastraporn Kaewpawong, Kiatkamjorn Kusol, Hasanah Pairoh

**Affiliations:** 1 Excellence Center of Community Health Promotion, School of Nursing, Walailak University, Nakhon Si Thammarat 80160, Thailand; pastraporn3@gmail.com; 2 Department of Professional Nursing Studies, Kulliyyah of Nursing, IIUM, Kuantan Campus, Kuantan 25200, Pahang, Malaysia

**Keywords:** situation, factors, dental caries, preschool children

## Abstract

**Highlights:**

**What are the main findings?**

• The prevalence of dental caries was 52.6%, and 55.6% of children had visible oral uncleanliness.
• Caregiver oral health care practices, caregiver education, and dental visits for toothache were independently associated with dental caries.

**What are the implications of the main findings?**

• Dental caries remains common among preschool children.
• Oral health promotion should strengthen caregivers’ practical oral health care skills and promote preventive dental care.

**Abstract:**

Background: Early childhood caries (ECC) remains a major public health concern and is influenced by interactions among children, environmental, and dental care factors. Purpose: This study aimed to determine the prevalence of dental caries and identify factors associated with dental caries among preschool children in Nakhon Si Thammarat Province. Methods: A cross-sectional study was conducted among 435 children aged 3–5 years and their primary caregivers. Oral health was assessed by preliminary visual screening, and caregivers completed structured questionnaires on demographic characteristics, oral health attitudes and practices, and dental service utilization. Generalized estimating equations (GEEs) were used to account for clustering within child development centers. Results: Dental caries was identified in 52.6% of children, and 55.6% had visible oral uncleanliness. In multivariable GEE analysis, higher caregiver oral health care practice scores were associated with lower odds of dental caries (AOR = 0.05; 95% CI: 0.02–0.14; *p* < 0.001). Children whose caregivers had an educational level below a bachelor’s degree had higher odds of dental caries (AOR = 3.03; 95% CI: 2.09–4.41; *p* < 0.001). Children who were never or only sometimes taken to a dentist for toothache also had higher odds of dental caries (AOR = 1.95; 95% CI: 1.31–2.90; *p* = 0.001). Conclusions: Dental caries remains common among preschool children in southern Thailand. Strengthening caregivers’ practical oral health care skills and promoting preventive rather than symptom-driven dental care may support caries prevention.

## 1. Introduction

Early childhood caries (ECC) represents a major public health challenge worldwide. According to the World Health Organization (WHO), more than 353 million children worldwide are affected by dental caries [1]. As the most prevalent non-communicable disease among young children, ECC has an average global prevalence of approximately 49% [2]. Its occurrence is influenced by multiple interacting biological, behavioral, socioeconomic, and structural factors [3,4,5,6]. Southeast Asia has one of the highest reported prevalence rates, reaching up to 89% in some areas [2,7]. In Thailand, the prevalence of ECC remains high at 75.6%, underscoring that dental caries remains a major public health concern requiring effective prevention and control strategies [2].

The findings of the 9th National Oral Health Survey further emphasize the magnitude of this problem. Approximately 38.0% of 3-year-old children and 45.4% of 5-year-old children had early-stage dental caries. More importantly, untreated caries was found in 46.1% of 3-year-olds and increased to 70.4% among 5-year-olds. In addition, more than 40% of preschool children had visible dental plaque, placing them at high risk for developing new carious lesions [8]. The consequences of ECC extend beyond oral health. Dental pain may impair chewing, leading to selective eating that reduces dietary quality and increases the risk of malnutrition. Previous studies in Thailand have also reported that preschool children with dental caries have a 4.9 times higher risk of iron-deficiency anemia than children without caries [9]. In addition, chronic oral infection associated with untreated dental caries may contribute to systemic health problems and adversely affect children’s quality of life, family well-being, school attendance, and healthcare expenditure [1,10,11].

Southern Thailand remains one of the regions with the highest burden of dental caries. The prevalence among 3–5-year-old children has been reported at 55.5% and 79.6%, respectively, with higher rates observed in rural areas than in urban communities [8]. A similar pattern has been reported in Nakhon Si Thammarat Province, where 52.9% of 3-year-old children had dental caries and a large proportion presented with visible dental plaque, indicating a high risk of disease progression [8]. Community oral health assessments have consistently classified the province as a high-risk area for dental caries. Furthermore, provincial surveillance data in 2025 showed that only 49% of preschool children received oral health screening, which remained below the national target [8,12]. These findings suggest that existing preventive strategies have not yet achieved sufficient coverage or effectiveness in this setting.

The WHO framework on the determinants of health proposes that health outcomes are influenced not only by individual characteristics but also by environmental and health system factors [4,6,13]. Guided by this framework, the present study considered three domains potentially associated with dental caries among preschool children. Child-related factors, bottle-feeding practices, and oral hygiene behaviors, including toothbrushing frequency, duration, and caregiver assistance during toothbrushing, have consistently been associated with ECC [8,12,14,15,16,17,18]. Regular toothbrushing with fluoride toothpaste is recognized as the primary strategy for controlling dental plaque and preventing dental caries [12,17]. However, because preschool children have limited manual dexterity, effective toothbrushing often depends on supervision and assistance from primary caregivers [18].

Environmental factors are equally important in shaping children’s oral health. The home environment, particularly caregivers’ educational level, oral health attitudes, and oral health care practices, plays a central role in establishing children’s daily oral hygiene and dietary behaviors [8,19,20]. At the same time, child development centers provide an important setting where teachers’ oral health care practices for preschool children can reinforce healthy oral hygiene habits during the daytime [20,21]. Together, these homes and environments may influence children’s oral health behaviors and subsequent risk of dental caries.

At the health system level, equitable access to and utilization of preventive dental services, including routine dental examinations and fluoride application, are essential components of ECC prevention [4,6]. Despite the proven effectiveness of these preventive measures, disparities in access to and utilization of oral health services remain common, particularly in rural communities, potentially limiting their impact on reducing dental caries burden.

Although previous studies in Thailand have identified several factors associated with ECC, most have focused primarily on child characteristics or caregiver-related factors [15,22]. Few studies have examined the combined influence of child, environmental, and health system-related factors within a single conceptual framework, particularly in southern Thailand. Therefore, this study aimed to determine the prevalence of dental caries and examine factors associated with dental caries among preschool children in Nakhon Si Thammarat Province. The findings may inform the development of integrated oral health promotion strategies involving families, child development centers, healthcare providers, and communities to improve oral health outcomes among preschool children.

### Research Conceptual Framework

The conceptual framework of this study was developed based on the World Health Organization (WHO) framework on the determinants of health [13]. This framework categorizes the independent variables into three domains: (1) child-related factors, including bottle-feeding practices and oral hygiene behaviors (frequency and duration of toothbrushing); (2) environmental factors, comprising caregivers’ oral health attitudes and care practices in the home environment, together with teachers’ oral health care practices for preschool children in child development centers; and (3) health system factors, including access to and utilization of dental services. These domains were hypothesized to be associated with the dependent variable, dental caries among preschool children. The conceptual framework guided the selection of study variables and the examination of factors associated with dental caries in Nakhon Si Thammarat Province, Thailand.

## 2. Materials and Methods

### 2.1. Study Design

This cross-sectional study aimed to determine the prevalence of dental caries and identify factors associated with dental caries among preschool children in Nakhon Si Thammarat Province, Thailand. Data were collected from May to July 2025.

### 2.2. Measures

The study population consisted of 45,346 children aged 3–5 years in Nakhon Si Thammarat Province [23]. The sample size was calculated using Krejcie and Morgan’s (1970) formula, yielding a sample of 435 participants [24].

Setting the sample size by Krejcie and Morgan’s formula:

From $n=\frac{X^{2}Np(1-p)}{e^{2}(N-1)\;+X^{2}p(1-p)}$

When

n = Sample size

N = Population size

e = Acceptable level of errors

X^2^ = Chi-square with df = 1 and 95% reliability (X^2^ = 3.841)

p = Proportion of interested characteristics in the population (In case of unknown, set p = 0.5)

Representation $\begin{array}{l} n\;=\frac{3.841(45,346)(0.5)(1-0.5)}{0.05^{2}(45,346)+3.841(0.5)\;(1-0.5)}\\ \;\;\;=434.19\;samples\;\left(sample\;size=435\right) \end{array}$

A total of 435 samples were obtained in this research.

The required sample size for this study was initially calculated to be 435 participants. To account for possible attrition and incomplete responses, an additional 10% was added to the calculated sample size. Consequently, the final sample comprised 479 participants.

Participants were selected using a multistage sampling technique. Nakhon Si Thammarat Province consists of 23 districts. In the first stage, four districts—Mueang, Tha Sala, Pak Phanang, and Thung Yai—were randomly selected. In the second stage, one subdistrict was selected from each district. Child development centers were subsequently selected within each selected area. Two child development centers were included from Mueang, Tha Sala, and Thung Yai districts, whereas three centers were included from Pak Phanang District because the number of eligible preschool children in each center was relatively small. Thus, a total of nine child development centers were included. Within each center, eligible children were selected using simple random sampling, with the number of participants allocated according to the number of eligible children in each center

The inclusion criteria for this study were children aged 3 to 5 years enrolled in Child Development Centers in Nakhon Si Thammarat Province. Their caregivers were required to be proficient in reading and writing Thai and must have provided voluntary informed consent for participation. Conversely, the exclusion criteria applied to children with physical disabilities or severe systemic conditions that could impact oral health or dental formation, such as cleft lip and palate, Down syndrome, thalassemia, or active cancer treatment. Additionally, children who were absent on data-collection dates or uncooperative during the oral health examination were excluded from the study. In the end, the minimum required sample size was 435 participants. An additional 10% was initially allowed for potential non-response or incomplete data, resulting in a recruitment target of up to 479 participants. During the data-collection period, 435 eligible child–caregiver pairs were successfully recruited and provided complete data. Therefore, all 435 participants with complete data were included in the final analysis.

### 2.3. Research Instruments

Research instruments were detailed as follows:

Part 1: Demographic Questionnaire. This part included questions on the demographic characteristics of both children (weight, height, date of birth, age, and gender) and their caregivers (gender, education level, and relation to the child). It also collected information on children’s oral hygiene practices, feeding practices, and access to and utilization of dental services. The questionnaire consisted of both closed-ended and open-ended questions.

Part 2: Children’s oral health status. Oral health status was assessed through preliminary visual screening, informed by the oral health assessment framework described in the Report of the 9th National Oral Health Survey of Thailand [8], with modifications appropriate for the community-based setting. The researchers, who were pediatric nursing instructors, visually inspected the children’s teeth using a small handheld flashlight in a well-lit area within the child development center. No dental mirror, probe, air-drying, or other dental instruments were used. The screening recorded visible dental caries, filled teeth, and oral cleanliness. Oral cleanliness was recorded as clean or unclean without using a standardized oral hygiene index. Dental caries was recorded when a visible carious lesion was identified; non-cavitated early enamel lesions were not systematically assessed. Prior to data collection, the examiners received standardized training from dentists and dental personnel; however, formal inter- and intra-examiner reliability coefficients were not assessed.

Part 3: Caregivers’ attitudes toward children’s oral health. This section consisted of a 10-item questionnaire adapted from relevant literature and the Report of the 9th National Oral Health Survey of Thailand [8]. A 5-point Likert scale was employed, ranging from 5 (strongly agree) to 1 (strongly disagree). The instrument included both positive and negative items, with reverse scoring applied to the latter. Mean scores were categorized into three levels: high (3.67–5.00), moderate (2.34–3.66), and low (1.00–2.33).

Part 4: Caregivers’ oral health care practices for preschool children. This section comprised a 16-item questionnaire adapted from relevant literature and the Report of the 9th National Oral Health Survey of Thailand [8]. It assessed caregivers’ oral health care practices over the previous 6 months, including oral hygiene, dietary, and bottle-feeding practices, as well as utilization of dental services. Responses were rated on a four-point Likert scale ranging from 4 (regularly) to 1 (never). Mean scores were categorized into three levels: high (3.01–4.00), moderate (2.01–3.00), and low (1.00–2.00).

Part 5: Teachers’ oral health care practices for children. This section consisted of a 9-item questionnaire developed based on relevant literature and the Report of the 9th National Oral Health Survey of Thailand [8]. It evaluated teachers’ oral health care practices over the previous 6 months, including oral hygiene practices and dietary supervision. Responses were rated on a four-point Likert scale ranging from 4 (regularly) to 1 (never). Mean scores were categorized as high (3.01–4.00), moderate (2.01–3.00), or low (1.00–2.00).

Regarding the quality of the research instruments, content validity was evaluated by a panel of five experts comprising two pediatric nursing specialists and three community nursing instructors. The content validity index (CVI) obtained demonstrated strong alignment with the theoretical framework and study objectives. Specifically, the CVI values for caregivers’ attitudes, caregivers’ oral health care behaviors, and teachers’ oral health care behaviors were 0.96, 0.97, and 1.00, respectively.

The refined questionnaires were pilot-tested with 30 caregivers and teachers with characteristics similar to those of the target population. Internal consistency was assessed using Cronbach’s alpha coefficient. The Cronbach’s alpha coefficients were 0.79 for caregivers’ attitudes, 0.78 for caregivers’ oral health care practices, and 0.80 for teachers’ oral health care practices, respectively.

### 2.4. Data Collection

After the research proposal was approved by the Human Research Ethics Committee of Walailak University, permission to conduct the study was formally obtained from the relevant institutions.

1. The researchers wrote a letter of request for support from the School of Nursing, Walailak University, to the directors of all child development centers included in the study.
2. The researchers contacted the directors of all child development centers, parents, and research participants to clarify the project details, objectives, processes, and expected benefits. They also confirmed that their participation must be based on their consent. The researchers also requested their kind assistance to collect all data.
3. The researchers studied the early childhood sample in Nakhon Si Thammarat Province, where 3–5-year-olds in child development centers were randomly selected.
4. The researchers collected data by handing the questionnaire to all participants at child development centers in the community, with a detailed description of each question. Children were selected from the list of early childhood students by simple random sampling until the required number was reached. The participants had 30–45 min to complete the questionnaire.
5. The researchers examined the accuracy and completeness of all data.

### 2.5. Ethical Statement

The researchers conducted this study in accordance with the principles outlined in the Declaration of Helsinki. All procedures involving human participants adhered to the ethical standards of the relevant institutional board. Approval for this study was obtained from the Ethics Committee on Human Research at Walailak University on 26 February 2025 (WUEC-25-070-01), as mandated prior to data collection. Informed consent was obtained from all participants and their parents and/or guardians before they were included in the study. The children aged 3–5 years and their parents will provide written consent. Research information will be stored securely, with data coded for confidentiality.

### 2.6. Statistical Analysis

Statistical analyses were performed using IBM SPSS Statistics for Windows, Version 24.0 (IBM Corp., Armonk, NY, USA). Descriptive statistics included frequencies, percentages, means, and standard deviations (SDs). For inferential analyses, caregiver attitude, caregiver oral health care practice, and teacher oral health care practice scores were treated as continuous variables. Generalized estimating equations (GEEs) with a binomial distribution, logit link function, exchangeable working correlation structure, and robust standard errors were used to account for clustering of children within the nine child development centers. Univariable GEE models were used to estimate crude odds ratios (CORs), followed by multivariable GEE analysis to estimate adjusted odds ratios (AORs). Variables were included in the multivariable model based on conceptual relevance and findings from the univariable analyses. Multicollinearity among predictors was assessed using variance inflation factors (VIFs). CORs and AORs with 95% confidence intervals (CIs) were reported, with *p* < 0.05 considered statistically significant.

## 3. Results

Among the 435 children included in the study, 52.6% had dental caries. More than half of the children (55.6%) were classified as having visibly poor oral cleanliness based on the visual screening. The mean age of participants was 3.83 years (S.D. = 0.71), with 4-year-old children representing the largest age group (44.1%). Most children had a normal nutritional status (78.2%). The majority of primary caregivers were parents (81.1%), and over half had an educational level below a bachelor’s degree (56.1%), as displayed in Table 1.

Regarding home and child development center environmental factors, Caregivers’ attitudes toward children’s oral health (Mean = 3.99, S.D. = 0.71), oral hygiene care practices (Mean = 3.07, S.D. = 0.55), and bottle-feeding practices (Mean = 3.19, S.D. = 0.85), whereas dietary practices were at a moderate level (Mean = 2.72, S.D. = 0.43). Teachers’ oral health care practices for children were at a high level (Mean = 3.66, S.D. = 0.21), as shown in Table 2.

Overall access to and utilization of dental services were at a moderate level (mean = 2.05, S.D. = 0.99). The highest mean score was observed for dental visits because of toothache (mean = 2.68, S.D. = 1.07), followed by fluoride application (mean = 2.09, S.D. = 1.01), routine six-month dental check-ups (mean = 2.07, S.D. = 0.99), and dental fillings (mean = 2.03, S.D. = 1.12). Dental visits for tooth extraction and never having visited a dentist had the lowest mean scores, as shown in Table 3.

The GEE bivariate analysis showed that several factors were significantly associated with dental caries. Children who brushed their teeth for less than one minute had higher odds of dental caries than those who brushed for at least one minute (COR = 1.80, 95% CI: 1.04–3.14, *p* = 0.037). Similarly, children who brushed independently had higher odds of dental caries than those who received caregiver assistance (COR = 1.83, 95% CI: 1.22–2.75, *p* = 0.004). Bottle feeding and brushing once daily were not significantly associated with dental caries (*p* > 0.05).

Caregiver-related factors were also associated with dental caries. Children whose caregivers had an educational level below a bachelor’s degree had three times the odds of dental caries compared with those whose caregivers had a bachelor’s degree or higher (COR = 3.00, 95% CI: 2.20–4.10, *p* < 0.001). Higher caregiver oral health attitude and oral health care practice scores were associated with lower odds of dental caries (COR = 0.36, 95% CI: 0.20–0.65, *p* = 0.001; and COR = 0.04, 95% CI: 0.01–0.15, *p* < 0.001, respectively). Teachers’ oral health care practice scores were not significantly associated with dental caries (*p* = 0.064).

Dental service-related factors were significantly associated with dental caries. Children who never or only sometimes visited a dentist for toothache, received six-month dental check-ups, or received fluoride applications had higher odds of dental caries than those who received these services often or regularly (COR = 3.04, 95% CI: 2.35–3.94; COR = 3.12, 95% CI: 2.05–4.76; and COR = 2.68, 95% CI: 1.98–3.63, respectively; all *p* < 0.001), as shown in Table 4.

No evidence of problematic multicollinearity was observed among the predictors, with VIF values ranging from 1.096 to 1.781. In the multivariable generalized estimating equation (GEE) analysis, three variables remained significantly associated with dental caries. Each one-unit increase in the caregiver oral health care practice score was associated with substantially lower odds of dental caries (AOR = 0.05, 95% CI: 0.02–0.14, *p* < 0.001). Children whose caregivers had an educational level below a bachelor’s degree had approximately three times higher odds of dental caries than those whose caregivers had a bachelor’s degree or higher (AOR = 3.03, 95% CI: 2.09–4.41, *p* < 0.001). In addition, children who were never or only sometimes taken to a dentist for toothache had higher odds of dental caries than those who were taken often or regularly (AOR = 1.95, 95% CI: 1.31–2.90, *p* = 0.001), as shown in Table 5.

## 4. Discussion

This study aimed to examine the prevalence and factors associated with dental caries among preschool children in Nakhon Si Thammarat Province, Thailand. The findings showed that the prevalence of dental caries was 52.6%, which is classified as a very high level according to the criteria of the 9th National Oral Health Survey, where a community prevalence exceeding 50% is considered a major public health concern [8]. The prevalence observed in this study was lower than the national average (75.6%) and lower than that reported in several Southeast Asian countries [7,25]. Notably, more than half of the children (55.6%) were classified as having visibly poor oral cleanliness. This finding indicates that inadequate oral cleanliness was common among the preschool children in this study. The relatively high prevalence of visible oral uncleanliness, together with the observed prevalence of dental caries, highlights the importance of strengthening daily oral hygiene practices among preschool children, including regular toothbrushing and appropriate caregiver supervision [17,18].

### Factors Associated with Dental Caries Among Preschool Children

Among child-related factors, brushing duration and toothbrushing assistance were significantly associated with dental caries in the univariable analysis. Children who brushed for less than one minute and those who brushed independently had higher odds of dental caries than their respective reference groups. However, these associations were no longer statistically significant after adjustment for other variables. Bottle-feeding status and toothbrushing frequency were also not independently associated with dental caries in the multivariable analysis. These findings suggest that the observed associations with brushing characteristics may be influenced by broader caregiving practices, particularly caregivers’ routine oral hygiene behaviors. Previous studies have reported that brushing teeth at least twice daily is associated with a lower prevalence of dental caries among preschool children [4,26,27]. Regular toothbrushing helps remove dental plaque and maintain fluoride on tooth surfaces, thereby reducing acid production by cariogenic bacteria. Similar observations have been reported by Duangthip et al. (2017), who suggested that the effectiveness and consistency of tooth cleaning may have a greater influence on dental caries than brushing duration alone [7]. Nevertheless, adequate brushing facilitates more effective plaque removal because tooth surfaces are more likely to be cleaned thoroughly [12]. Therefore, caregivers should continue to follow recommendations to brush children’s teeth at least twice daily with fluoride toothpaste and provide supervision throughout the preschool years [12].

Within the home environment, caregiver educational level and oral health care practices were associated with dental caries among preschool children. Children whose primary caregivers had an educational level below a bachelor’s degree had approximately three times higher odds of dental caries than those whose caregivers had attained a bachelor’s degree or higher (AOR = 3.03, 95% CI: 2.09–4.41). This finding is consistent with previous studies reporting that caregiver education is associated with children’s oral health through better health literacy, greater access to health information, and an improved ability to adopt appropriate preventive practices [19,28,29]. Caregivers with higher educational attainment may also be more likely to establish regular oral hygiene routines and seek preventive dental care for their children.

A notable finding of this study was that caregivers’ oral health care practice scores remained strongly associated with children’s dental caries after adjustment for other variables. Higher caregiver oral health care practice mean scores were associated with lower odds of dental caries (AOR = 0.05, 95% CI: 0.02–0.14). This finding is biologically plausible because preschool children have limited manual dexterity and are not yet able to clean their teeth effectively without adult supervision [12]. Daily caregiver involvement in toothbrushing, supervision of fluoride toothpaste use, and regulation of sugary food and beverage consumption therefore remain essential components of caries prevention. These findings emphasize the importance of strengthening caregivers’ practical skills and confidence in providing daily oral health care, particularly among families with lower educational attainment.

Interestingly, although higher caregiver oral health attitude scores were significantly associated with lower odds of dental caries in the univariable analysis, this association was no longer statistically significant after adjustment for other variables. This finding suggests that positive attitudes alone may not necessarily translate into appropriate oral health practices. Similar observations have been reported by Naidu et al. (2020), who found that although many caregivers recognized the importance of children’s oral health, they experienced difficulties implementing recommended preventive practices, including supervising toothbrushing, limiting sugary snacks, and seeking preventive dental care [30]. Although caregivers demonstrated generally favorable oral health attitudes, positive attitudes may not always translate into consistent daily oral health practices. In particular, young children may require consistent caregiver supervision and assistance to maintain appropriate toothbrushing practices. Therefore, oral health promotion programs should focus not only on improving caregivers’ knowledge and attitudes but also on strengthening practical caregiving skills and supporting consistent supervision of children’s daily oral hygiene routines [17,18].

Within child development centers, teachers’ oral health care practices for preschool children were not significantly associated with children’s dental caries. Although teachers reported a high level of oral health care practices, these practices were not independently associated with children’s dental caries. One possible explanation is that oral health behaviors established in child development centers account for only part of children’s daily oral health care. Preschool children spend a substantial proportion of their time at home, where toothbrushing routines, dietary habits, and caregiver supervision are more consistently maintained. Consequently, the influence of caregiver practices may outweigh that of teacher-supervised oral health care during childcare hours.

This finding is consistent with the multivariable analysis, in which caregiver oral health care practices remained significantly associated with dental caries, whereas teacher-supervised oral health care practices were not significantly associated with the outcome. These findings highlight the importance of continuity between home and child development center environments. Previous studies have similarly suggested that oral health promotion activities conducted in educational settings may have a limited impact when they are not reinforced by consistent oral health practices at home [31,32]. Nevertheless, child development centers remain an important setting for establishing healthy oral hygiene habits during early childhood. Daily supervised toothbrushing after meals, reducing the availability of sugary snacks, and strengthening communication between teachers and caregivers may help reinforce oral health messages delivered in both settings. Therefore, oral health promotion programs should encourage stronger collaboration among caregivers, teachers, and local health professionals to ensure that preventive practices are consistently implemented throughout children’s daily lives.

Access to and utilization of dental services were also associated with dental caries among preschool children. Children who were never or only sometimes taken to a dentist for toothache had 1.95 times higher odds of dental caries than those who were taken often or regularly (AOR = 1.95, 95% CI: 1.31–2.90). One possible explanation is that caregivers’ care-seeking behavior may be symptom-driven rather than prevention-oriented. Caregivers may delay dental visits until children experience pain or visible dental problems, resulting in dental attendance occurring after caries has already developed. In the univariable analysis, children who never or only sometimes received routine six-month dental check-ups or fluoride applications also had higher odds of dental caries. However, these associations were no longer statistically significant after adjustment for other variables. Similar findings have been reported in studies from China and Sri Lanka, where children with a history of dental visits often had a higher prevalence of dental caries because dental services were sought primarily for treatment rather than prevention [4,27]. Another possible explanation is that the apparent protective effects of routine dental check-ups and fluoride application observed in the bivariate analysis were attenuated after adjustment for child-, caregiver-, and dental service-related factors. This finding suggests that daily home oral health practices may have a greater influence on children’s oral health than preventive dental services alone. Preventive services remain important; however, their effectiveness is likely to depend on consistent caregiver participation in daily oral hygiene practices and appropriate dietary control. Therefore, oral health promotion strategies should encourage caregivers to seek regular preventive dental care before symptoms develop while simultaneously strengthening oral health practices within the home environment.

## 5. Conclusions

Dental caries remain common among preschool children in Nakhon Si Thammarat Province. After accounting for sampling within child development centers and adjusting for child-, caregiver-, and dental service-related factors, caregiver education level, caregiver oral health care practices, and symptom-driven dental attendance remained independently associated with dental caries. These findings highlight the importance of strengthening caregivers’ practical oral health care skills and promoting preventive rather than symptom-driven dental care. Collaboration among families, child development centers, and primary healthcare providers is important for improving oral health during early childhood.

## 6. Limitations

The oral health assessment was based on preliminary visual screening without dental mirrors, probes, air-drying, or other dental instruments. Therefore, early or non-cavitated lesions may have been missed, potentially underestimating dental caries prevalence. The standardized dmft index was not assessed, and formal inter- and intra-examiner reliability coefficients were not available, which may have introduced variability in the assessment. The cross-sectional design precludes causal inference, and residual confounding may remain despite multivariable adjustment. Caregiver- and teacher-reported practices may also be subject to recall and social desirability bias. Finally, although clustering within child development centers was accounted for using GEEs, the original sample-size calculation did not incorporate a design effect, which may have affected the precision of the estimates.

## Figures and Tables

**Table 1:** General information on children and their primary caregivers (n = 435).

Characteristics	Number	%
**Preschool children**		
Gender		
Female	229	52.6
Male	206	47.4
Age (years) (Mean 3.83, S.D = 0.714)		
3	165	38.0
4	192	44.1
5	78	17.9
Nutritional status (weight-for-height)		
Undernutrition (<−1.5 SD)	15	3.4
Normal (−1.5 SD to +1.5SD)	340	78.2
Overnutrition (>+1.5 SD)	80	18.4
Dental Caries		
Caries-free	206	47.4
Have dental caries	229	52.6
Oral cleanliness		
Clean	193	44.4
Unclean	242	55.6
**Primary caregiver**		
Gender		
Female	337	77.5
Male	98	22.5
Relationship to the child		
Father or mother	353	81.1
Grandparent/relative	82	18.9
Education level		
Below a bachelor’s degree	244	56.1
Bachelor’s degree or higher	191	43.9

**Table 2:** Mean scores and levels of home and child development center environmental factors (n = 435).

Environmental Factors	Mean	S.D.	Level
**Home environment**			
Caregivers’ attitudes toward children’s oral health	3.99	0.706	High
Caregivers’ oral health care practices for children			
- Oral hygiene practices	3.07	0.552	High
- Dietary practices	2.72	0.426	Moderate
- Bottle-Feeding Practices	3.19	0.852	High
**Child development center environment**			
Teachers’ oral health care practices for children	3.66	0.211	High

**Table 3:** Mean scores and levels of access to and utilization of dental services (n = 435).

Access and Utilization of Dental Services	Mean	S.D.	Level
1. Dental visit for toothache	2.68	1.074	Moderate
2. 6-month dental check-up	2.07	0.985	Moderate
3. Fluoride application	2.09	1.006	Moderate
4. Dental fillings	2.03	1.115	Moderate
5. Tooth extraction	1.75	0.858	Low
6. Never visited a dentist	1.72	0.882	Low
Overall	2.05	0.986	Moderate

**Table 4:** Univariable generalized estimating equation analyses of factors associated with dental caries among preschool children (n = 435).

Factors	COR	95% CI	*p*-Value
**Child-related factors**			
Bottle feeding: Bottle-fed vs. non-bottle-fed	1.76	0.96–3.24	0.069
Toothbrushing: Once/day vs. ≥2 times/day	2.89	0.95–8.76	0.061
Brushing duration: <1 min vs. ≥1 min	1.80	1.04–3.14	0.037
Independent brushing vs. caregiver assistance	1.83	1.22–2.75	0.004
**Caregiver-related factors**			
Education below bachelor’s vs. ≥bachelor’s	3.00	2.20–4.10	0.001
Caregiver oral health attitude score *	0.36	0.20–0.65	0.001
Caregiver oral health care practice score *	0.04	0.01–0.15	0.001
**Child development center-related factor**			
Teacher oral health care practice score *	2.33	0.95–5.70	0.064
**Dental service-related factors**			
Dental visit for toothache: Never/sometimes vs. often/regularly	3.04	2.35–3.94	<0.001
6-month dental check-up: Never/sometimes vs. often/regularly	3.12	2.05–4.76	<0.001
Fluoride application: Never/sometimes vs. often/regularly	2.68	1.98–3.63	<0.001

* Analyzed as a continuous mean score. COR = crude odds ratio; CI = confidence interval.

**Table 5:** Multivariable generalized estimating equation analysis of factors associated with dental caries among preschool children (n = 435).

Variable	B	AOR	95% CI	*p*-Value
Bottle feeding: Bottle-fed vs. non-bottle-fed	−0.146	0.86	0.55–1.36	0.528
Toothbrushing: Once/day vs. ≥2 times/day	0.662	1.94	0.54–6.95	0.309
Brushing duration: <1 min vs. ≥1 min	0.258	1.29	0.65–2.59	0.465
Independent brushing vs. caregiver assistance	0.291	1.34	0.74–2.41	0.332
Caregiver education below bachelor’s vs. ≥bachelor’s	1.110	3.03	2.09–4.41	0.001
Caregiver oral health attitude score *	0.262	1.30	0.81–2.09	0.278
Caregiver oral health care practice score *	−2.989	0.05	0.02–0.14	0.001
Teacher oral health care practice score *	1.308	3.70	0.95–14.44	0.060
Dental visit for toothache: Never/sometimes vs. often/regularly	0.666	1.95	1.31–2.90	0.001
6-month dental check-up: Never/sometimes vs. often/regularly	0.303	1.35	0.67–2.74	0.400
Fluoride application: Never/sometimes vs. often/regularly	0.245	1.28	0.95–1.72	0.105

* AOR = adjusted odds ratio; CI = confidence interval.

## Data Availability

The original contributions presented in this study are included in the article. Further inquiries can be directed to the corresponding author.
